# Provider Initiated Testing and Counseling (PITC) for HIV in resource-limited clinical settings: important questions unanswered

**DOI:** 10.4314/pamj.v3i1.52442

**Published:** 2009-09-04

**Authors:** Joseph Becker, Landry Tsague, Ruben Sahabo, Peter Twyman

**Affiliations:** 1 Stanford University School of Medicine, Department of Medicine, Division of Emergency Medicine,; 2 International Center for AIDS Care and Treatment Programs (ICAP), Columbia University, Kigali, Rwanda.

**Keywords:** Provider Initiated Testing and Counseling, PITC, Africa

## Editorial

HIV testing and counseling is the critical entry-point for engagement into treatment and care as well as for primary and secondary prevention efforts. Despite the importance of this step, most HIV-infected patients globally, and particularly in resource-poor settings, are unaware of their HIV status and uptake of Voluntary Counseling and Testing (VCT) services is relatively limited [1, 2]. Furthermore, data suggest that many patients receive their HIV diagnosis at a late stage in the disease after multiple clinical visits and contacts with the health care system, which likely represent missed opportunities for counseling and testing [3, 4]. Without effective approaches to routinely provide HIV counseling and testing, HIV-infected patients are likely to be identified at advanced stages of immunosuppression, when therapeutic response may be sub-optimal [5]. Initiatives such as the World Health Organization (WHO) “3 by 5” initiative, the Presidential Emergency Plan for AIDS Relief (PEPFAR) and the Global Fund to Fight AIDS, Tuberculosis and Malaria (GFATM), have sought to expand access to antiretroviral medications (ARV) in resource-poor settings including sub-Saharan Africa, the region hardest hit by the HIV epidemic [6].

However, in 2006 survey data from several sub-Saharan African countries suggested that only 10% of men and 12% of women had been tested for HIV, implying that the majority of HIV-infected persons in this region are unaware of their status. [7] In response to these realities there has been recent focus on the part of WHO, United States governmental agencies such as the Centers for Disease Control and Prevention (CDC) and the United States Agency for International Development (USAID), and numerous non-governmental organizations on increasing the accessibility of testing and making HIV testing a more ‘routine’ part of general medical care. While voluntary counseling and testing generally relies on the patient initiating attendance at a testing site, the ‘routine’ offer of HIV testing in health facilities, also termed Provider Initiated Testing and Counseling (PITC), uses the care provider to make a clinical recommendation that the patient have a voluntary HIV test. While PITC is meant to complement other testing strategies such as VCT and community based testing (CBT), critical differences remain which render PITC unique and limit extrapolation. Furthermore, routine PITC in general clinical settings poses several questions that have not been answered sufficiently such as 1) How will patients react to the routine offer of an HIV test? Will clinic attendance decline in order to avoid testing, and will providers accept the added burden of routine testing in their facilities? 2) Will routine PITC lead to a reduction in the number of ‘missed opportunities’ to identify HIV-infected patients and increase the number of those linked into care and treatment? 3) Are there ethical implications of PITC such as, how voluntary is HIV testing in these settings? Are there negative ramifications of routine PITC (i.e., intimate partner violence)? 4) What lessons learned from over 20 years of experience with VCT can assist policy makers and program managers in routine PITC implementation and scale up? These largely unanswered questions require further research to refine future routine PITC programs.

There is limited data regarding patient perceptions of routine PITC offered in general medical facilities. Several studies have documented high patient acceptance, however it is unclear to what extent this is an accurate surrogate for favorable patient perception, since intentional and unintentional coercion, as well as other factors, may confound a patient’s decision making at the point of testing [8, 9]. Between November 2004 and February 2006, Mbarara and Mulago teaching hospitals in Mbarara and Kampala, Uganda, respectively, offered routine testing to inpatients and their family members in participating medical and surgical units. The study revealed a test acceptance rate of 98%, and 81% of those tested had not been tested previously. HIV prevalence in those accepting testing was 25%. Among 1,213 couples tested, 224 (19%) had discordant testing results, which represents a significant opportunity for prevention, in addition to the opportunity to offer treatment to the HIV-infected member of the couple [10].

A survey in Botswana conducted in 2006 sought to describe the knowledge and attitudes of the general population approximately 11 months after the institution of a country-wide national policy to offer routine PITC. A total of 81% of respondents reported being very much or extremely in favor of routine HIV testing in health facilities, and 89% believed that the policy would decrease barriers to testing and improve the link to ARV treatment. Among the survey respondents previously tested through VCT or PITC, 93% indicated that they had decided to be tested on their own and 98% reported not regretting their decision to be tested. However, these positive results were tempered by the finding that 43% of those surveyed believed that the policy might lead to individuals avoiding their doctors for fear of testing [11]. The concern that aggressive testing in health facilities will decrease care seeking in general has been voiced since the early days of PITC and remains largely unaddressed. Indeed, there is no data documenting health facility attendance after PITC roll out and very little documentation of actual patient and community perceptions of particular PITC programs, or their willingness to present for general medical care. Furthermore, there is only very limited data describing the perceptions of providers regarding the feasibility and appropriateness of routine testing and counseling in their facilities and their willingness to participate in these programs.

The main justification for routine PITC is to increase the number of patients tested and thus the number of HIV-infected patients identified and linked to medical care and support services. In 2004, Botswana was the first African nation to introduce PITC in a widespread and systematic fashion. Data from the first two and a half years of the program revealed a dramatic increase in testing: from 60,846 being tested in 2004, to 157,894 in 2005, and to 88,218 in 2006. Testing rates in the population through this program were 40 per 1,000 persons, 93 per 1,000 persons, and 104 per 1,000 persons, per year respectively [8]. Similarly, in western Kenya, an emergency department-based routine PITC program demonstrated a 97% testing acceptance rate (1331/1379) with 82% of HIV infected patients attending their first post-test follow up clinic visit. A total of 312 HIV-infected persons (22.7% prevalence) were identified during the 5-month period of this study [11]. In central Haiti, Partners in Health instituted PITC at a primary care center to reduce missed opportunities to identify HIV-infected persons. Subsequent to the institution of this program 3,787 tests were performed and 112 newly diagnosed HIV-infected patients were identified via PITC targeted at patients with risk factors for HIV infection or a clinical presentation consistent with HIV infection. A total of 85% of the HIV-infected patients were identified on their first visit, leading us to hypothesize that the majority of these patients would not have initially been diagnosed prior to PITC and that this program accelerated the pathway to care and support [12].

Many authors have expressed concerns that routine opt-out approaches to HIV testing pose human rights challenges [13, 14, 15]. A critical ethical foundation to HIV testing, and indeed all medical interventions, is the ability of patients to provide informed consent. Informed consent stems from the concept of autonomy whereby patients have the right of self-determination, to act as agents for their own good, balancing their own individual costs and benefits regarding the decision to test. In the context of HIV/AIDS, where people living with HIV (PLHIV) face stigma and the threat of discrimination, social/familial rejection and even violence, these principles are particularly important. Given the substantial social status that health providers hold in many societies, there is concern that patients are either intentionally or unintentionally coerced at the point of testing and cannot really opt-out of PITC. Indeed, it is difficult to interpret the success of opt-out programs where the vast majority of patients agree to be tested. Is this an indication of program success or coercive methods of obtaining consent? Indeed, in the above survey regarding routine opt-out testing in Botswana, while the majority of respondents reported that routine testing was beneficial, 68% felt that they could not refuse a test offered by their provider [11].

As important as the ability to consent, is the need for this consent to be informed. In order to make routine testing more feasible in health facilities, WHO has recommended a streamlined approach to pre-test counseling involving processes such as group pre-test counseling, recorded or posted HIV educational materials, and improved community sensitization and education regarding HIV testing in general [3]. This guideline, while essential to routine HIV testing in busy clinical settings, has sparked discussion that improved testing numbers are being used to justify an erosion in the quality of pre-test counseling [13]. Integrating such a streamlined approach to pre-test counseling into a rushed, busy, and overwhelmed clinical setting may cause some concern regarding the quality of pre-test counseling in these settings. This predicted decline in the quality of pretest counseling is also cited as a further threat to informed consent as patients are theoretically provided with less information, particularly regarding the potential negative consequences of testing [13]. Data in this regard are extremely limited and therefore care must be taken to safeguard and ensure adequate patient education and knowledge prior to the offer of testing. In addition, research is needed to better understand informed consent in the context of PITC and to evaluate the most effective approaches.

HIV testing, and in particular disclosure, has been associated with multiple negative outcomes, including feelings of isolation and depression, the threat of ruptures of confidentiality, discrimination at home or in the workplace, and intimate partner and family violence [16]. Little is known regarding the frequency and severity of these outcomes which are undoubtedly dependent on a multitude of societal, cultural, and economic factors. In a study in Botswana, only 1% of those polled (who had been previously tested) indicated that their test had resulted in violence, 2% reported discrimination, and 5% a breach of healthcare worker confidentiality. However, 10% of the general respondent pool stated that their reason for not being tested was fear of partner violence, 11% feared discrimination by healthcare providers, and 18% feared rupture of confidentiality. A further 14% believed that routine testing would increase violence against women [11]. It is hypothesized that as routine testing becomes more common place, and as the availability of antiretroviral therapy increases, HIV stigma will decline and intimate partner violence and discrimination will also decrease [16, 17]. However, little data regarding the incidence of these outcomes currently exist to guide programming, and it is unknown to what degree HIV stigma, which is still significant in many societies, will abate as testing is more prominently promoted by medical practitioners.

Led by WHO and donors, efforts are underway to significantly expand PITC. In May 2007, WHO released its revised recommendations with regards to routine offer HIV testing in health facilities [3]. These guidelines provide some recommendations in terms of providing routine testing in health facilities. However, little operational data and experience have been generated to assist in PITC programming and the operational difficulties discussed above, such as if patients will avoid clinics offering routine PITC and what could ameliorate this possibility.

While scale up of access to HIV testing is critical, an ambitious research agenda should accompany this effort in order to answer key questions and to test the effectiveness of various intervention models. In addition, while the general public appears to have a positive perception of routine testing initiatives, data is limited and large-scale surveys or focus group discussions with patients who have participated in routine PITC efforts have not been undertaken. There has been little discussion regarding potential means of addressing the perceived lack of voluntariness of testing and methods for limiting coercion. While it is claimed that expanded treatment and testing will reduce stigma, there is little evidence to support this claim. Furthermore, there are many operational questions regarding implementation of PITC programs. How do PITC programs institute effective counseling and testing in busy and hurried clinical centers where time and privacy are often at a premium? When resources are limited and testing and counseling cannot be offered to all patients attending health facilities, how should testing be targeted? What patient information, symptomatology, and clinical signs are most predictive of HIV infection? While many approaches are in use, none have been validated or compared. Lastly, but importantly, how are patients identified via PITC programs best linked with treatment and support programs? These issues will be of central importance as new PITC programs are launched and existing one scale up.

Testing is the gateway to HIV care and support services, and efforts to broaden treatment must include a proactive and inclusive approach to testing. However, multiple challenges and questions remain regarding the provision of routine HIV testing and counseling in clinical facilities and it is important that testing programs be effectively integrated into routine provider activities, so as not to overburden already under-resourced providers and facilities. Furthermore, testing must be conducted in a manner which limits coercion and emphasizes voluntariness, and ensures effective linkages to care and treatment for those who test HIV positive. A research agenda that addresses the ethical and social ramifications of PITC, with a special focus on the operational components of PITC programming in health facilities, is critically needed to further guide its expansion.
